# Supplementary material for: Development of a computer-aided design software for the quantitative evaluation of aesthetic damage
Source: PLoS One. 2019 Dec 18;14(12):e0226322. doi: 10.1371/journal.pone.0226322 (PMC6919621; doi:10.1371/journal.pone.0226322)
Supplement: S1 Code site — 1. (ZIP) [file pone.0226322.s001.zip › [EESC_jr][Projeto]Relatorio_07_21/reports/templates_zip/gentelella-master.zip!/gentelella-master/vendors/DateJS/reports/lcov-report/core/core.js.html]

Code coverage report for core/core.js


# Code coverage report for core/core.js

## Statements: 96.77% (120 / 124)      Branches: 90.11% (82 / 91)      Functions: 91.18% (31 / 34)      Lines: 98.36% (120 / 122)      Ignored: none

All files » core/ » core.js

```
|  |  |  |
| --- | --- | --- |
| 1 2 3 4 5 6 7 8 9 10 11 12 13 14 15 16 17 18 19 20 21 22 23 24 25 26 27 28 29 30 31 32 33 34 35 36 37 38 39 40 41 42 43 44 45 46 47 48 49 50 51 52 53 54 55 56 57 58 59 60 61 62 63 64 65 66 67 68 69 70 71 72 73 74 75 76 77 78 79 80 81 82 83 84 85 86 87 88 89 90 91 92 93 94 95 96 97 98 99 100 101 102 103 104 105 106 107 108 109 110 111 112 113 114 115 116 117 118 119 120 121 122 123 124 125 126 127 128 129 130 131 132 133 134 135 136 137 138 139 140 141 142 143 144 145 146 147 148 149 150 151 152 153 154 155 156 157 158 159 160 161 162 163 164 165 166 167 168 169 170 171 172 173 174 175 176 177 178 179 180 181 182 183 184 185 186 187 188 189 190 191 192 193 194 195 196 197 198 199 200 201 202 203 204 205 206 207 208 209 210 211 212 213 214 215 216 217 218 219 220 221 222 223 224 225 226 227 228 229 230 231 232 233 234 235 236 237 238 239 240 241 242 243 244 245 246 247 248 249 250 251 252 253 254 255 256 257 258 259 260 261 262 263 264 265 266 267 268 269 270 271 272 273 274 275 276 277 278 279 280 281 282 283 284 285 286 287 288 289 290 291 292 293 294 295 296 297 298 299 300 301 302 303 304 305 306 307 308 309 310 311 312 313 314 315 316 317 318 319 320 321 322 323 324 325 326 327 328 329 330 331 332 333 334 335 336 337 338 339 340 341 342 343 344 | 1 1     15 15   15     1 1               1   1                       5 1 1   4 2     5 4 3   1       5 1 3                       5 2       1             1 2094             1 4                 1 408 5 403 401   2                   1 398               1 2               1 26 26 105 24     2               1 24 24 163 23     1               1 2               1 591                 1 588 12 12   588     1       1 6 6 166 166 5       1     1 7 7 7 399 8     7 1   6 5   1 2 1           1 15 15 15     1 2 2 2 2       1 369 369 1 368 1 367       28   339               1 1               1 3               1 5               1 30               1 119 119               1 1               1 139               1                     71   1 3 3   1   2 | ``` (function () { 	var $D = Date, 		$P = $D.prototype, 		p = function (s, l) { 			Eif (!l) { 				l = 2; 			} 			return ("000" + s).slice(l * -1); 		}; 	 	Eif (typeof window !== "undefined" && typeof window.console !== "undefined" && typeof window.console.log !== "undefined") { 		$D.console = console; // used only to raise non-critical errors if available 	} else { 		// set mock so we don't give errors. 		$D.console = { 			log: function(){}, 			error: function(){} 		}; 	} 	$D.Config = $D.Config \|\| {};   	$D.initOverloads = function() { 		/**  		 * Overload of Date.now. Allows an alternate call for Date.now where it returns the  		 * current Date as an object rather than just milliseconds since the Unix Epoch. 		 * 		 * Also provides an implementation of now() for browsers (IE<9) that don't have it. 		 *  		 * Backwards compatible so with work with either: 		 *  Date.now() [returns ms] 		 * or 		 *  Date.now(true) [returns Date] 		 */ 		if (!$D.now) { 			$D._now = function now() { 				return new Date().getTime(); 			}; 		} else if (!$D._now) { 			$D._now = $D.now; 		}   		$D.now = function (returnObj) { 			if (returnObj) { 				return $D.present(); 			} else { 				return $D._now(); 			} 		};   		if ( !$P.toISOString ) { 			$P.toISOString = function() { 				return this.getUTCFullYear() + 				"-" + p(this.getUTCMonth() + 1) + 				"-" + p(this.getUTCDate()) + 				"T" + p(this.getUTCHours()) + 				":" + p(this.getUTCMinutes()) + 				":" + p(this.getUTCSeconds()) + 				"." + String( (this.getUTCMilliseconds()/1000).toFixed(3)).slice(2, 5) + 				"Z"; 			}; 		} 		 		// private 		if ( $P._toString === undefined ){ 			$P._toString = $P.toString; 		}   	}; 	$D.initOverloads();     	/**  	 * Gets a date that is set to the current date. The time is set to the start of the day (00:00 or 12:00 AM). 	 * @return {Date}    The current date. 	 */ 	$D.today = function () { 		return new Date().clearTime(); 	};   	/**  	 * Gets a date that is set to the current date and time (same as new Date, but chainable) 	 * @return {Date}    The current date. 	 */ 	$D.present = function () { 		return new Date(); 	};   	/** 	 * Compares the first date to the second date and returns an number indication of their relative values.   	 * @param {Date}     First Date object to compare [Required]. 	 * @param {Date}     Second Date object to compare to [Required]. 	 * @return {Number}  -1 = date1 is lessthan date2. 0 = values are equal. 1 = date1 is greaterthan date2. 	 */ 	$D.compare = function (date1, date2) { 		if (isNaN(date1) \|\| isNaN(date2)) { 			throw new Error(date1 + " - " + date2); 		} else if (date1 instanceof Date && date2 instanceof Date) { 			return (date1 < date2) ? -1 : (date1 > date2) ? 1 : 0; 		} else { 			throw new TypeError(date1 + " - " + date2); 		} 	}; 	 	/** 	 * Compares the first Date object to the second Date object and returns true if they are equal.   	 * @param {Date}     First Date object to compare [Required] 	 * @param {Date}     Second Date object to compare to [Required] 	 * @return {Boolean} true if dates are equal. false if they are not equal. 	 */ 	$D.equals = function (date1, date2) { 		return (date1.compareTo(date2) === 0); 	};   	/** 	 * Gets the language appropriate day name when given the day number(0-6) 	 * eg - 0 == Sunday 	 * @return {String}  The day name 	 */ 	$D.getDayName = function (n) { 		return Date.CultureInfo.dayNames[n]; 	};   	/** 	 * Gets the day number (0-6) if given a CultureInfo specific string which is a valid dayName, abbreviatedDayName or shortestDayName (two char). 	 * @param {String}   The name of the day (eg. "Monday, "Mon", "tuesday", "tue", "We", "we"). 	 * @return {Number}  The day number 	 */ 	$D.getDayNumberFromName = function (name) { 		var n = Date.CultureInfo.dayNames, m = Date.CultureInfo.abbreviatedDayNames, o = Date.CultureInfo.shortestDayNames, s = name.toLowerCase(); 		for (var i = 0; i < n.length; i++) { 			if (n[i].toLowerCase() === s \|\| m[i].toLowerCase() === s \|\| o[i].toLowerCase() === s) { 				return i; 			} 		} 		return -1; 	}; 	 	/** 	 * Gets the month number (0-11) if given a Culture Info specific string which is a valid monthName or abbreviatedMonthName. 	 * @param {String}   The name of the month (eg. "February, "Feb", "october", "oct"). 	 * @return {Number}  The day number 	 */ 	$D.getMonthNumberFromName = function (name) { 		var n = Date.CultureInfo.monthNames, m = Date.CultureInfo.abbreviatedMonthNames, s = name.toLowerCase(); 		for (var i = 0; i < n.length; i++) { 			if (n[i].toLowerCase() === s \|\| m[i].toLowerCase() === s) { 				return i; 			} 		} 		return -1; 	};   	/** 	 * Gets the language appropriate month name when given the month number(0-11) 	 * eg - 0 == January 	 * @return {String}  The month name 	 */ 	$D.getMonthName = function (n) { 		return Date.CultureInfo.monthNames[n]; 	};   	/** 	 * Determines if the current date instance is within a LeapYear. 	 * @param {Number}   The year. 	 * @return {Boolean} true if date is within a LeapYear, otherwise false. 	 */ 	$D.isLeapYear = function (year) { 		return ((year % 4 === 0 && year % 100 !== 0) \|\| year % 400 === 0); 	};   	/** 	 * Gets the number of days in the month, given a year and month value. Automatically corrects for LeapYear. 	 * @param {Number}   The year. 	 * @param {Number}   The month (0-11). 	 * @return {Number}  The number of days in the month. 	 */ 	$D.getDaysInMonth = function (year, month) { 		if (!month && $D.validateMonth(year)) { 				month = year; 				year = Date.today().getFullYear(); 		} 		return [31, ($D.isLeapYear(year) ? 29 : 28), 31, 30, 31, 30, 31, 31, 30, 31, 30, 31][month]; 	};   	$P.getDaysInMonth = function () { 		return $D.getDaysInMonth(this.getFullYear(), this.getMonth()); 	};   	$D.getTimezoneAbbreviation = function (offset, dst) { 		var p, n = (dst \|\| false) ? Date.CultureInfo.abbreviatedTimeZoneDST : Date.CultureInfo.abbreviatedTimeZoneStandard; 		for (p in n) { 			Eif (n.hasOwnProperty(p)) { 				if (n[p] === offset) { 					return p; 				} 			} 		} 		return null; 	}; 	 	$D.getTimezoneOffset = function (name, dst) { 		var i, a =[], z = Date.CultureInfo.timezones; 		Iif (!name) { name = (new Date()).getTimezone();} 		for (i = 0; i < z.length; i++) { 			if (z[i].name === name.toUpperCase()) { 				a.push(i); 			} 		} 		if (!z[a[0]]) { 			return null; 		} 		if (a.length === 1 \|\| !dst) { 			return z[a[0]].offset; 		} else { 			for (i=0; i < a.length; i++) { 				if (z[a[i]].dst) { 					return z[a[i]].offset; 				} 			} 		} 	};   	$D.getQuarter = function (d) { 		d = d \|\| new Date(); // If no date supplied, use today 		var q = [1,2,3,4]; 		return q[Math.floor(d.getMonth() / 3)]; // ~~~ is a bitwise op. Faster than Math.floor 	};   	$D.getDaysLeftInQuarter = function (d) { 		d = d \|\| new Date(); 		var qEnd = new Date(d); 		qEnd.setMonth(qEnd.getMonth() + 3 - qEnd.getMonth() % 3, 0); 		return Math.floor((qEnd - d) / 8.64e7); 	};   	// private 	var validate = function (n, min, max, name) { 		name = name ? name : "Object"; 		if (typeof n === "undefined") { 			return false; 		} else if (typeof n !== "number") { 			throw new TypeError(n + " is not a Number."); 		} else if (n < min \|\| n > max) { 			// As failing validation is *not* an exceptional circumstance  			// lets not throw a RangeError Exception here.  			// It's semantically correct but it's not sensible. 			return false; 		} 		return true; 	};   	/** 	 * Validates the number is within an acceptable range for milliseconds [0-999]. 	 * @param {Number}   The number to check if within range. 	 * @return {Boolean} true if within range, otherwise false. 	 */ 	$D.validateMillisecond = function (value) { 		return validate(value, 0, 999, "millisecond"); 	};   	/** 	 * Validates the number is within an acceptable range for seconds [0-59]. 	 * @param {Number}   The number to check if within range. 	 * @return {Boolean} true if within range, otherwise false. 	 */ 	$D.validateSecond = function (value) { 		return validate(value, 0, 59, "second"); 	};   	/** 	 * Validates the number is within an acceptable range for minutes [0-59]. 	 * @param {Number}   The number to check if within range. 	 * @return {Boolean} true if within range, otherwise false. 	 */ 	$D.validateMinute = function (value) { 		return validate(value, 0, 59, "minute"); 	};   	/** 	 * Validates the number is within an acceptable range for hours [0-23]. 	 * @param {Number}   The number to check if within range. 	 * @return {Boolean} true if within range, otherwise false. 	 */ 	$D.validateHour = function (value) { 		return validate(value, 0, 23, "hour"); 	};   	/** 	 * Validates the number is within an acceptable range for the days in a month [0-MaxDaysInMonth]. 	 * @param {Number}   The number to check if within range. 	 * @return {Boolean} true if within range, otherwise false. 	 */ 	$D.validateDay = function (value, year, month) { 		Iif (year === undefined \|\| year === null \|\| month === undefined \|\| month === null) { return false;} 		return validate(value, 1, $D.getDaysInMonth(year, month), "day"); 	};   	/** 	 * Validates the number is within an acceptable range for months [0-11]. 	 * @param {Number}   The number to check if within range. 	 * @return {Boolean} true if within range, otherwise false. 	 */ 	$D.validateWeek = function (value) { 		return validate(value, 0, 53, "week"); 	};   	/** 	 * Validates the number is within an acceptable range for months [0-11]. 	 * @param {Number}   The number to check if within range. 	 * @return {Boolean} true if within range, otherwise false. 	 */ 	$D.validateMonth = function (value) { 		return validate(value, 0, 11, "month"); 	};   	/** 	 * Validates the number is within an acceptable range for years. 	 * @param {Number}   The number to check if within range. 	 * @return {Boolean} true if within range, otherwise false. 	 */ 	$D.validateYear = function (value) { 		/** 		 * Per ECMAScript spec the range of times supported by Date objects is  		 * exactly -100,000,000 days to +100,000,000 days measured relative to  		 * midnight at the beginning of 01 January, 1970 UTC.  		 * This gives a range of 8,640,000,000,000,000 milliseconds to either  		 * side of 01 January, 1970 UTC. 		 * 		 * Earliest possible date: Tue, 20 Apr 271,822 B.C. 00:00:00 UTC 		 * Latest possible date: Sat, 13 Sep 275,760 00:00:00 UTC 		 */ 		return validate(value, -271822, 275760, "year"); 	}; 	$D.validateTimezone = function(value) { 		var timezones = {"ACDT":1,"ACST":1,"ACT":1,"ADT":1,"AEDT":1,"AEST":1,"AFT":1,"AKDT":1,"AKST":1,"AMST":1,"AMT":1,"ART":1,"AST":1,"AWDT":1,"AWST":1,"AZOST":1,"AZT":1,"BDT":1,"BIOT":1,"BIT":1,"BOT":1,"BRT":1,"BST":1,"BTT":1,"CAT":1,"CCT":1,"CDT":1,"CEDT":1,"CEST":1,"CET":1,"CHADT":1,"CHAST":1,"CHOT":1,"ChST":1,"CHUT":1,"CIST":1,"CIT":1,"CKT":1,"CLST":1,"CLT":1,"COST":1,"COT":1,"CST":1,"CT":1,"CVT":1,"CWST":1,"CXT":1,"DAVT":1,"DDUT":1,"DFT":1,"EASST":1,"EAST":1,"EAT":1,"ECT":1,"EDT":1,"EEDT":1,"EEST":1,"EET":1,"EGST":1,"EGT":1,"EIT":1,"EST":1,"FET":1,"FJT":1,"FKST":1,"FKT":1,"FNT":1,"GALT":1,"GAMT":1,"GET":1,"GFT":1,"GILT":1,"GIT":1,"GMT":1,"GST":1,"GYT":1,"HADT":1,"HAEC":1,"HAST":1,"HKT":1,"HMT":1,"HOVT":1,"HST":1,"ICT":1,"IDT":1,"IOT":1,"IRDT":1,"IRKT":1,"IRST":1,"IST":1,"JST":1,"KGT":1,"KOST":1,"KRAT":1,"KST":1,"LHST":1,"LINT":1,"MAGT":1,"MART":1,"MAWT":1,"MDT":1,"MET":1,"MEST":1,"MHT":1,"MIST":1,"MIT":1,"MMT":1,"MSK":1,"MST":1,"MUT":1,"MVT":1,"MYT":1,"NCT":1,"NDT":1,"NFT":1,"NPT":1,"NST":1,"NT":1,"NUT":1,"NZDT":1,"NZST":1,"OMST":1,"ORAT":1,"PDT":1,"PET":1,"PETT":1,"PGT":1,"PHOT":1,"PHT":1,"PKT":1,"PMDT":1,"PMST":1,"PONT":1,"PST":1,"PYST":1,"PYT":1,"RET":1,"ROTT":1,"SAKT":1,"SAMT":1,"SAST":1,"SBT":1,"SCT":1,"SGT":1,"SLST":1,"SRT":1,"SST":1,"SYOT":1,"TAHT":1,"THA":1,"TFT":1,"TJT":1,"TKT":1,"TLT":1,"TMT":1,"TOT":1,"TVT":1,"UCT":1,"ULAT":1,"UTC":1,"UYST":1,"UYT":1,"UZT":1,"VET":1,"VLAT":1,"VOLT":1,"VOST":1,"VUT":1,"WAKT":1,"WAST":1,"WAT":1,"WEDT":1,"WEST":1,"WET":1,"WST":1,"YAKT":1,"YEKT":1,"Z":1}; 		return (timezones[value] === 1); 	}; 	$D.validateTimezoneOffset= function(value) { 		// timezones go from +14hrs to -12hrs, the +X hours are negative offsets. 		return (value > -841 && value < 721); 	};   }());   ``` |
```

Generated by istanbul at Sun Nov 16 2014 12:08:33 GMT-0800 (PST)
